# Trends, challenges, and opportunities for the United States alternative meat and seafood sector: stakeholder-informed perspectives

**DOI:** 10.1038/s41538-026-00841-4

**Published:** 2026-04-17

**Authors:** Amanda Wood, Katherine Consavage Stanley, Jack Daly, Khara Grieger, William R. Aimutis, Rohan A. Shirwaiker, Norbert L. W. Wilson

**Affiliations:** 1 Department of Energy and Technology, Swedish University of Agricultural Sciences, Uppsala, Sweden; 2 Bezos Center for Sustainable Protein at North Carolina State University, Raleigh, NC USA; 3 World Food Policy Center, Sanford School of Public Policy, Duke University, Durham, NC USA; 4 Department of Applied Ecology, North Carolina State University, Raleigh, NC USA; 5 Edward P. Fitts Department of Industrial & Systems Engineering, North Carolina State University, Raleigh, NC USA

**Keywords:** Business and management, Business and management, Economics, Economics, Environmental social sciences, Information systems and information technology

## Abstract

Growing global protein demand has fueled innovation and investment in alternative protein (AP) products, including plant-based, fermentation-derived, and cell-cultivated products. Through interviews with AP stakeholders in the United States (U.S.), we explored the sector’s evolution, challenges, opportunities, and trends. Interviewees described a boom from 2009 to 2021 followed by a decline, which the sector is now working to reverse. Achieving taste and price parity, attracting a broad consumer base, producing at scale, and navigating a charged policy environment remain key sector challenges. Looking forward, stakeholders were optimistic, noting opportunities including collaborations within and across sectors; workforce development; innovative financing, scalability models, and products; and increased policy engagement. Findings indicate that the U.S. AP sector is at a critical inflection point. This study points to future research, financing, and innovation that could help AP products become a mainstay in the food system.

## Introduction

Demand for protein is increasing globally due to a growing world population, shifting demographics to older populations where protein requirements are higher, and rising incomes leading to increased demand of animal-source foods^1,2^. In response, the scientific community, food industry, media, and the public have paid considerable attention to alternative protein (AP) products as a potential solution to help meet this growing demand, particularly in the United States (U.S.) and other high-income countries^3,4^. The term “AP products” refers to plant-based, fermentation-derived, and cell-cultivated protein products that mimic the sensory characteristics of traditional meat and seafood products.

This increased global protein demand has resulted in a spur of innovation within the AP sector in recent decades, drawing on technological developments in other sectors, such as the biomedical and pharmaceutical industries^5^. For instance, novel fermentation methods (e.g., precision fermentation) that use microbes and plants to produce protein have been developed^3,6^, and cell-cultivated proteins, which are made from animal cells grown in a food production facility, have emerged.

AP innovation has been boosted by growing investments in the past decade. For instance, from 2014 to 2021, AP funding grew exponentially, reaching a peak of $5.6 billion in global investment in 2021^6^. In the past 5 years, the AP sector secured the first U.S. regulatory approvals for cell-cultivated products^7^, and the plant-based market has experienced substantial growth in product diversity, although plant-based AP sales have declined since 2022^8,9^. Interest in APs has also increased due to the potential environmental, human health, and animal wellbeing benefits of APs over conventional animal-based foods^4,10–12^.

Despite considerable innovation, investment, and attention towards alternative meat and seafood products in the past decade, the sector is now facing challenges. Notably, there have been declines in investment, in part due to a broader reduction in food tech funding, investor shifts to other areas (e.g., artificial intelligence)^9,13^, waning consumer interest^14^, and macroeconomic shifts like higher interest rates leading to more risk-adverse investments^15^. In addition, the sector has faced difficulties reaching commercial scale, navigating evolving policy landscapes, and challenges in attaining widespread consumer acceptance in the U.S.^4,6,7,16–19^.

Research has been conducted to explore the market^20,21^, consumer^16,22,23^, and policy^7^ landscapes for much of the AP sector. However, few published studies have captured insights from U.S. AP stakeholders on the current status and perspectives of these products and their future potential^24,25^. This study thus fills a gap in the literature by describing the market, consumer, policy, and stakeholder landscapes for AP meat and seafood products in the U.S. based on expert perceptions.

This research study provides a timely analysis of stakeholder perspectives on challenges and opportunities for the U.S. AP sector, as of mid-2025. The stakeholders interviewed for this study spanned the AP landscape in expertise, representing AP start-ups, industry associations, investors, government regulators, civil society organizations (referred to non-governmental organizations [NGOs] for this study), philanthropies, and academia. Insights from stakeholders can inform research agendas and spur innovations that enhance the ability of the AP sector—alongside traditional protein sources—to deliver protein-rich foods with social, environmental, and economic benefits. Additionally, insights from this research can inform innovation theories and their applicability to these emerging technologies.

## Results

In total, 21 stakeholders were interviewed between January and May 2025. One interviewee (P16) opted out of the study after the interview, so their data was withdrawn prior to data analysis. This led to a total of 20 stakeholder interviews included in this analysis. Table 1 provides an overview of participating stakeholders. We sought to include stakeholders with a wide range of perspectives. However, participation was voluntary, and individuals and entities that have publicly expressed less favorable views of AP products, such as some in the conventional agriculture sector, declined to participate (see “Methods”). Therefore, the results obtained in this study may not fully reflect the perspectives of individuals and entities who are more critical of AP products.

**Table 1:** Study participant characteristics (*n* = 20)

Policy & government (*n* = 2)	Companies (*n* = 3)	Industry associations (*n* = 2)	Investors (*n* = 4)	Non-governmental organizations & philanthropies (*n* = 3)	Research (*n* = 6)
1. National regulator 2. National regulator	1. Multinational protein company 2. Alternative protein (AP) seafood startup 3. AP seafood startup	1. Food industry association 2. Plant-based industry association	1. Food venture capitalist (VC) 2. Food investment advisory firm 3. Food VC 4. Food VC	1. AP nonprofit 2. AP nonprofit 3. AP nonprofit	1. University AP center 2. University AP center 3. AP research expert 4. AP research expert 5. AP research expert 6. AP research expert

Insights from AP stakeholder interviews are presented below. We first share stakeholder reflections on the evolution of the sector to date and the breadth of individuals and entities engaged in the AP sector. We then discuss stakeholders’ perspectives on the characteristics of AP consumers and the challenges and opportunities for commercialization and policy. We describe the consumer, policy, and industry actor narratives that emerged from stakeholder reflections on the sector, along with relevant stakeholder dynamics that were mentioned. Lastly, we reflect on participants’ visions for the role of APs in the future food system. This structure follows closely with the interview guide in Supplementary File 1.

Stakeholders were free to discuss the AP sector as a whole or focus on specific AP types (i.e., plant-based, fermented, and cell-cultivated). We aim to preserve stakeholder terminology as much as possible. Thus, it was not always possible to determine to which specific AP type(s) stakeholders referred. Some participants associated a given insight with a particular AP, while others discussed it in relation to APs more generally. We also note that fermented AP products—encompassing traditional, biomass, and precision fermentation—represent a diverse category that was rarely mentioned explicitly. However, we believe that some stakeholders included such products within the broader category of plant-based APs, since fermented ingredients are often components of these products (e.g., the fermented heme iron, leghemoglobin, used in Impossible Foods’ plant-based burger^26^). Direct quotes include the deidentified participant number, presented in the format (P#).

### Evolution of the AP sector

Stakeholders described an explosion of activity and interest in the AP sector between 2009 and 2021 followed by a period of declining momentum. One described this as “the venture capital boom and bust (P3).” Another likened AP’s trajectory to the Gartner Hype Cycle (GHC) innovation framework (see “Discussion”).

Stakeholders noted several key events that led to the AP boom. Beyond Meat and Impossible Foods were recognized as market leaders at the time, launching their U.S. products in 2012 and 2016, respectively. These products “had something new to bring to the table (P8),” notably, their focus on mimicking the sensory properties of meat. For example, in Impossible Foods’ products, “heme proteins gave these products more of a meaty flavor when you cook them, and consumer interest skyrocketed for a few years (P4).” It was also a significant period globally for cell-cultivated meat following the development of the first cell-cultivated burger in 2013 by a Dutch researcher, Dr. Mark Post. From a funding perspective, “We saw a huge boom and a lot of venture capital going into the alt protein space (P3).” Fueling the funding craze, Beyond Meat launched an initial public offering in May 2019, which “catalyzed about $15 billion… in the form of private investments between 2020 and 2025 (P19)” for the AP sector.

Stakeholders noted that the plant-based AP sector grew rapidly between 2019 and 2021 before reaching a “bubble in 2021 and 2022 (P19).” The COVID-19 pandemic contributed to this inflection point for APs already on the market. Stakeholders described this as a time when a large influx of AP products on the market coincided with the need for people to eat meals at home, resulting in increased AP sales. Stakeholders offered several hypotheses for the increase: people cooked more to pass the time, interest in health increased, or perhaps supply chain issues catalyzed a need to vary food choices. However, “When people start to leave their homes and go back to their more regular or default lives and diets, much has collapsed (P6).” This post-pandemic downturn was attributed to multiple factors, including inflation, not meeting consumer taste or price expectations, concerns around APs as ultra-processed foods (UPFs), and market saturation. As one stakeholder reflected, the excitement around these products spurred “a whole range of ‘me too’ chicken nuggets, ‘me too’ burgers, which had no real innovation (P8),” and in turn, led to waning consumer interest.

For the cell-cultivated sector, although a few products had received U.S. regulatory approval at the time of interviews (meaning they could be sold in the U.S. market), participants described a loss of media and investor interest in recent years. This was in part due to the lack of products on the market, and in part to what was described as a slow, backlogged regulatory approval process in the U.S., discussed more below. Another offered, “There [were] a lot of companies started, a lot of great goals in mind, a lot of good initial technology, but products were not able to be made at a cost and scale to have any meaningful impact in the timeframes we’re talking about, so a lot of interest was lost because of that (P4).”

**Fig. 1:**
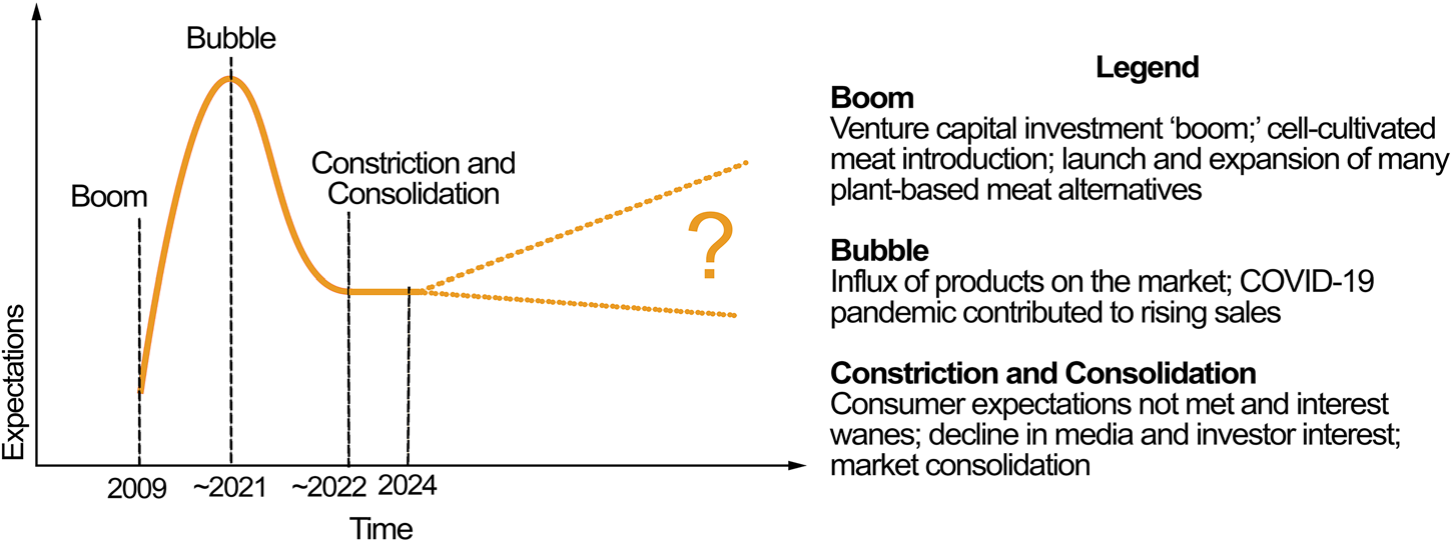
Key points on the evolution of the AP sector in the U.S., as described by study participants. This figure was adapted using a purchased license from iStock.com/PeterHermesFurian.

Describing the present state of AP innovation, one stakeholder said, “There’s been a bunch of constriction and consolidation across the world, which I think is inevitable as part of the histories of these new kinds of technologies (P6).” In fact, one stakeholder believed this period was good for the sector: “I think it was a good thing for the field to go through, in part because you had to rethink priorities and the pace of innovation to have a meaningful impact on consumers (P4).” However, one stakeholder believed that the AP sector was still struggling, noting, “I just think this year will be the rock bottom of the dissolution, and I really think [in] 2026, we’ll start climbing out of it (P19).” Figure 1 depicts the evolution of the U.S. AP sector to date, as described by our participants.

### Key stakeholders in the AP ecosystem

**Fig. 2:**
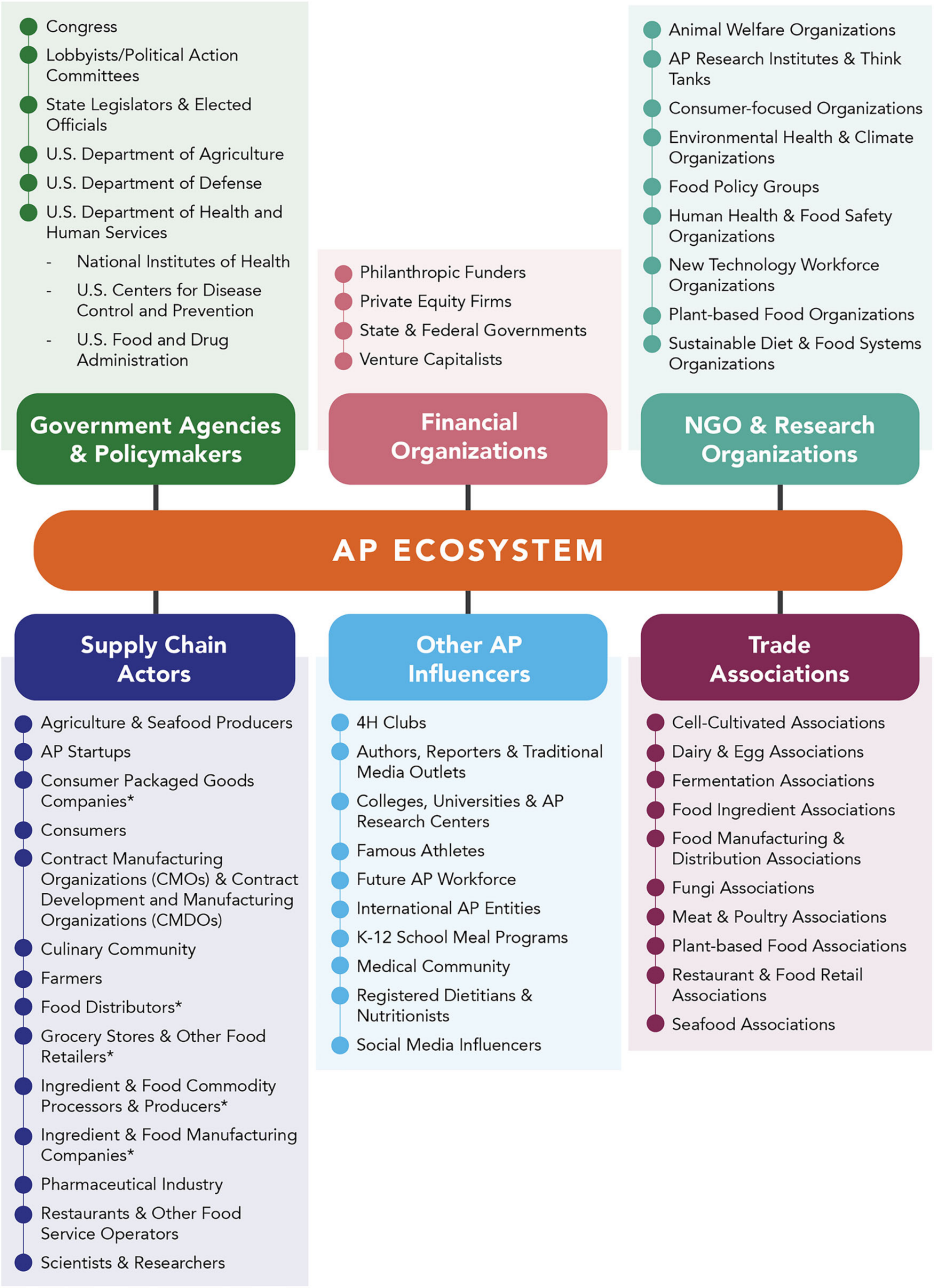
Current and emerging actors in the alternative protein (AP) ecosystem. The actors in the figure were identified by participants but categorized into six groups by the authors. Some actors or entities fit into multiple categories. Actors and entities have been listed alphabetically. Asterisk: Many participants described these organizations more broadly as “multinationals,” “big players,” or “legacy actors.” However, others mentioned organizations and entities within these categories, such as family-run grocery stores, that would not fit such descriptions, and thus, we chose the most representative and encompassing label available.

Interviewees identified a wide variety of entities engaged in the AP sector—including NGOs, trade associations, start-up companies, legacy food actors, universities, government agencies, politicians, and investment firms, among others. They also identified individuals and entities who could be more engaged to support scale-up, such as legacy food actors, or those who could advocate or influence the sector in the future, such as the medical community, nutritionists, restaurants and food service operators, chefs, food retailers, and social media influencers (Fig. 2). The current and potential future role of these entities is described in the following sections.

### Consumer landscape

The initial AP boom demonstrated that a broad consumer base was willing to try AP products. As one stakeholder described, “There’s been a huge revolution in how folks are actually thinking about what markets are alternative proteins intending to serve. This is no longer just the thing you toss on a barbecue for the vegetarians (P17).” Stakeholders estimated that vegans and vegetarians account for only 1–4% of the population, and while stakeholders recognized that some consumers might be compelled by animal welfare and environmental issues, others emphasized that these factors do not drive most consumers’ purchasing decisions. This led the AP sector to target other market segments, such as those “who want to maybe just decrease the amount of meat in their diet (P3).” Still for other “equal opportunity eaters (P3),” APs might “just be a choice they [consumers] want to make because they taste more delicious (P5).”

The increasingly diverse AP consumer base led many stakeholders to underscore the necessity of additional market research and crafting an enticing, inclusive consumer narrative, as discussed more below. Stakeholders were clear on one industry objective: AP products must deliver on taste and price. One stakeholder attributed the decline in repeat purchase to these two factors: “When you look at what consumers said, things largely fall into two buckets, which is this either: ‘didn’t wow me on taste’ …or, the cost went up, and the cost has become increasingly, obviously, a critical driver at a moment where you also had huge inflation around food (P5).” Many stakeholders felt that achieving taste parity with traditional meat products is not enough: AP products need to be the more delicious option. “…There’s just not a big enough fraction of consumers who are willing to pay more for a product that doesn’t taste as good as what they’re used to (P17).” The same goes for price – stakeholders expressed that AP products must be the cheaper option.

Several stakeholders mentioned that consumers may base their perceptions of the sector on just one AP tasting experience. An unsatisfactory sensory experience “sticks in their mind (P13)” regardless of how much the sector evolves. As a result, stakeholders felt that, in the AP sector, “The bar has been raised and everybody’s going to have to elevate to it (P12).” Another stressed, “The MVP – minimal viable product model – doesn’t really work for food because you don’t want to release food that’s not ready for prime time, or that’s not good enough, or that consumers don’t love (P5).”

Stakeholders identified nutrition and health as a “complicated area (P20)” of consumer concerns. Many stakeholders mentioned nutrition and health as consumer priorities. As one participant shared, “I think we’ve nailed to some extent the sensory part, but now…what is underneath it is the nutritional aspect of it. The question I feel like people are asking is, ‘Is it truly quote, unquote, “healthier” for me?’ (P19).” Another explained that consumers “have these overall health goals: increased protein, increased fiber, reduced cholesterol, and reduced saturated fat (P12).” Yet a few stakeholders shared their perspective that consumers would have different purchasing patterns (e.g., less junk food) if they prioritized nutrition.

From a broader health perspective, traditional seafood is a challenging area for certain consumers concerned with contaminants such as mercury, microplastics, environmental pollutants, pathogens, or parasites; stakeholders believed that cell-cultivated seafood could address these challenges. As one shared, “There’s so many problems you’re solving with cell-cultured seafood, particularly those that are big bio-accumulators (P1).” Another stakeholder expanded on the health benefits: “I think that’s an opportunity with cell ag, where we can come in and say: ‘Hey, it’s an alternative seafood that’s also still got that health halo that you’re looking for. It’s still going to be high in protein’ (P10)” and could contain omega-3 fatty acids. “Clean” eating is also on consumers’ radars, and one stakeholder explained, “A lot of shoppers talk about eating clean, less ingredients…Sometimes just seeing a shorter list is what consumers are looking for (P13).” “Clean” eating links to the concept of UPFs, another area where participants believed consumer attention is turning. One stakeholder explained, “Consumers do feel that [APs are] healthy, but even now with all this talk about UPF, that’s being questioned (P13).”

### Commercialization pathway

Participants touched on many aspects of commercialization, and below we focus only on the most frequently discussed areas across interviews. Stakeholders identified scaling as a core commercialization challenge for the sector; thus, the structure of the text below reflects the flow of the commercialization pathway rather than an order of priority. Stakeholders also described other countries’ AP innovations, which are beyond the scope of this paper. While regulatory considerations are relevant to commercialization, we explore those comments in the “Policy Landscape” section.

Focusing first on financing and foundational research and development (R&D), stakeholders emphasized the sheer extent of innovation needed to bring APs to market and its associated costs. As explained in the “Evolution of the sector” section, VCs were the primary early backers of many startups, yet VC funding has waned in recent years. One stakeholder explained, “So venture capital is down…it means it’s much harder to take risks, and for an R&D and alt protein specific point of view, I think it’s becoming clear that the private sector alone isn’t going to have the capital to underwrite the work that needs to happen (P6).” Given the retreat of VC funding and the need for capital expenditure (CapEx), i.e., money spent on long-term assets such as buildings and equipment, stakeholders emphasized the need for novel funding sources. Participants noted that government support will be necessary since “There’s no natural funder in the world that wants to take a large bet with a lot of CapEx and wants to do so with an emerging technology for an unproven market (P15).” This participant went on to explain what government funding could look like: “So things like loan guarantees, transferable tax credits, government procurement guarantees or commitments, those sorts of things are really important for scale up (P15).”

Despite a changing and challenging funding landscape, participants emphasized the progress made by the AP sector. For example, in the cell-cultivated space, “The science is advancing quickly and, frankly, faster than the amount of investment that’s gone into the space (P5).” Several participants used the first cell-cultivated burger to benchmark progress, emphasizing that the cost of a cell-cultivated burger has plummeted from over $300,000 in 2013 to about $100 in the 2025 market. One commented, “So we’ve come down orders of magnitude in cost of production in 10 years, even with little public funding support (P4).”

According to participants, the pace and progress of R&D differ by company and by AP category. R&D for plant-based APs is the most advanced, with products on the market for over a decade. Stakeholders described that R&D for plant-based APs is now focused on refining product offerings, such as improving sensory characteristics. Stakeholders mentioned that plant-based companies are using techniques like fiber spinning, 3D printing, and molds to produce more finished, structured products, such as whole filets. In contrast, cell-cultivated technology is “obviously still largely in the R&D phase (P5).” For at least one cell-cultivated seafood company, innovation was needed at every stage of the development and production process, including creating the technology to propagate cells and building a food-grade supply chain.

Second, focusing on scaling and infrastructure aspects of the commercialization pathway, participants underscored that scaling is a main challenge for the AP sector. While different AP types face distinct scale-up challenges, participants agreed that a shared challenge is the need to lower costs. “Alt proteins will have zero impact in the world unless they can achieve scale. And the only way they can achieve scale is if they’re really, really low cost (P15).” Another participant-identified challenge is the need for novel manufacturing facilities: “Traditional animal protein manufacturing is a disassembly business…This technology we are dealing with now, with either cultivated, fermentation, even plant-based, is an assembly business (P8),” which requires new manufacturing facilities. These facilities are expensive, and one stakeholder explained that “At the pilot scale, we see companies in the alt protein space maybe get into this spiral of raising funds and then changing their process and then raising more funds, and never quite graduating on to production scale (P3).” Another stakeholder shared their perspective that commercializing AP technology and running a manufacturing company were “very separate kinds of businesses (P15),” not to mention the expense required for one company to build demonstration or pilot scale facilities that may “fundamentally never be profitable in the market (P15).”

Given these challenges, the need for shared infrastructure and novel funding solutions to build that infrastructure was identified. One stakeholder explained the importance of engaging established food distributors as investors: “We’re not going to create our own distribution infrastructure. We have to leverage that of others…The more we get more strategic investors in the space here, that’s going to be helpful for all of us (P1).” Several stakeholders believed that the AP sector must attract Contract Manufacturing Organizations or Contract Development and Manufacturing Organizations—organizations that provide specialized manufacturing infrastructure and services to client companies on a contractual basis, enabling them to outsource production and accelerate scale-up.

Looking specifically at plant-based scaling challenges, even though “plant-based products [are] available at large scale in the market (P5),” few plant-based companies have been able to harness scale to lower costs. One stakeholder explained that cost-reduction was attainable only for “the mostly incumbent actors, the ones who have actually had products on the market for a number of years and have the ability to scale and, through scaling manufacturing, reduce costs to some degree (P20)”—namely Impossible Foods and Beyond Meat at that time. Another emphasized the need for collaboration in the scaling process: “I think the runway of throwing things against the wall and seeing what sticks is probably run out, and finding [what]… truly does deliver and then getting the partners that can help scale it, that’s where the value will start to be seen (P12).”

While precision fermentation and cell-cultivated APs are fundamentally different products, bioreactors are central to the manufacturing and scale-up challenges of both AP products. Again, the challenge of bringing these technologies to scale was emphasized: “At no time in our history have we taken cultivated meat and grown it at scale. It’s just never happened. Therefore, the first facility that is at scale needs to be designed, envisioned and [so] on (P8).” Stakeholders also explained that solving challenges at a smaller scale does not guarantee a smooth scale-up. For example, “It’s not necessarily the case that, if you’ve proven it at small scale, once you scale up, you’ll solve the price thing and you’re done. It might be that the technology needs to be different, that the way something performs in a much larger bioreactor is different (P5).” The same stakeholder pointed to future resource challenges, as more bioreactors will be needed, yet the materials to produce them are expensive and scarce. The need to test these products in larger bioreactors across the scale-up process again reflected the desire for shared infrastructure.

In an effort to avoid reinventing the wheel, stakeholders shared that cell-cultivated product companies have looked to the biopharmaceutical industry, since their experience seems closest to the scale-up challenges faced by APs using bioreactors. Some stakeholders, however, cautioned against borrowing too heavily from that industry: “…There’s nothing wrong with what the pharmaceutical industry created for scale-up and production processes, but I don’t see how this will work for the immense scales and low costs that we need for food-related products (P4).” Several stakeholders called instead for more innovation in production technology, with one emphasizing the need for “moonshot thinking (P18)” about how to scale cell-cultivated products. Several shared the need for advancement not only in manufacturing infrastructure, but also in techniques. For example, moving from batch manufacturing to continuous processes was noted as “the single biggest differentiator in terms of unit economics for the companies that we’ve looked at in precision fermentation, biomass fermentation, and cultivated (P15).”

Third, we focus on opportunities discussed by stakeholders for product development. Stakeholders mentioned products that were either combinations of different AP types or a mix of APs with a conventional meat source as one opportunity space. These products have been described as “hybrid” or “blended” proteins, and more recently as “balanced protein” by the AP sector. Referring to products composed of both APs and conventional meat, one stakeholder, who preferred the term “balanced proteins” for these products, emphasized their ability to reach taste parity: “…In blind sensory panels, some of these products outcompete 100% animal-based products purely on taste (P6).” According to participants, products that blend AP types might help cell-cultivated companies scale faster, especially since commercialized cell-cultivated products to date include a significant percentage of plant-based components. One stakeholder shared, “If you’re talking about cultivated meat in terms of a whole burger or a whole steak, the volume you have to produce is extraordinarily high. If you’re talking about 3% or 5% cultivated fat cells, it’s much more reasonable to think about. So, I think that’s a question mark, can you get these blends of plant-based plus cultivated fat that really get you across that taste gap? (P5).”

Fermented foods were also identified as an opportunity, since “the probiotic, prebiotic natures of fermented foods, the fact that technology’s already there, the fact that we’ve been fermenting foods forever and a day, all of this stuff makes it easier for the consumer to grab onto (P2).” Another suggested that product innovations should include ready-to-go meals, particularly those with international flavors. Stakeholders believed there was value in creating structured products (e.g., a whole filet instead of a nugget or burger) but that producing fully structured products is “technically challenging (P10).” AP seafood companies saw opportunities for growth in many seafood products, since seafood is already more expensive than some beef and poultry products, and “the unit economics make a lot more sense with seafood (P10).” While some have focused on lower-cost seafood, such as whitefish, stakeholders shared that other companies have focused on higher-end products like salmon, tuna, and sea bass, given high demand and higher price points. Across AP types, while some stakeholders see higher-value APs as the big opportunity space in the coming years, others see the opportunity in scale. One stakeholder reasoned, “If the alt protein industry’s goal is to move the needle on meat consumption in the U.S. or globally, it’s okay if people of means are the first to adopt these technologies (P3).” Yet they warned that the industry cannot claim to be “revolutionizing how meat is produced for the whole globe (P3)” until APs are affordable to all.

Fourth, several stakeholders noted the need to build a strong AP workforce. Some felt that the sector had already attracted passionate individuals. However, they expressed that more individuals with diverse backgrounds need to be recruited, such as, “…people who have more food industry experience; people who have more scaled manufacturing experience; people who have gone through scale up in other sectors and sort of understand the ways in which that can go wrong and go poorly; people with more commercial business strategy acumen (P15).” One stakeholder also mentioned the benefit of hiring individuals with a farming or agricultural background who might more easily engage with conventional agricultural actors. Others shared their belief that the AP sector should focus on building a workforce pipeline, including training college students and early-career professionals. That way, trained workers are available when innovations reach scale, as opposed to the first boom cycle, when skilled workers were in short supply.

### Policy landscape

At the time interviews were conducted, only two cell-cultivated meat companies (GOOD Meat and UPSIDE Foods) had completed the FDA-USDA regulatory review process (see Consavage Stanley & Kouivusaari et al. 2025^7^ for a description of this regulatory process). Some participants expressed optimism around the cell-cultivated regulatory process and the collaboration between cell-cultivated companies and government agencies to ensure product safety. As one shared, “Everyone wants to do it right, and they want to make sure, at the end of the day, the food we’re going to provide to consumers is safe and something that we can all stand behind…And consumers that worry about safety, you just look at the fact that these foods have to go through two different rounds of federal agency approval, not just one (P4).” However, others expressed concerns about what they described as a “backlog (P10)” of cell-cultivated products awaiting regulatory clearance, with one noting that some cell-cultivated products had been in the pipeline for 2 or 3 years. Another requested more clarity on the regulatory approval timeline. It is important to note that, in the months following these interviews, 3 additional cell-cultivated products completed regulatory review.

Stakeholders perceived that multiple policy-relevant actions were “wins” for the sector. These included state and federal government investments in AP R&D; the FDA’s draft labeling guidance for plant-based products that indicated consumers are not confused by AP product labeling; the development of the bipartisan Protein Innovation Caucus; and multiple state bills that have expanded access to plant-based products.

Conversely, nearly all participants described ongoing legislative challenges for the sector, especially for cell-cultivated products, in the form of labeling restrictions and bans on the production, sale, and/or distribution of these products. AP labeling restrictions have been around since early plant-based products started gaining popularity around 2016. Cell-cultivated bans have been more recent, and some stakeholders expressed that these bans were largely partisan. As one shared, “I mean you can look at it and it’s kind of following along political lines. More conservative states are putting forward bans and more liberal states haven’t touched the issue. Some have, but to a lesser extent. There’ve been proposed bans in some very blue states, but they’ve yet to go through (P10).”

A few stakeholders believed that the incumbent industry was behind these legislative challenges. Interviewees were confused by the rationale for the bans, in particular, since cell-cultivated products are not yet widely available to consumers. However, they shared their belief that protecting the conventional agriculture industry was a driver behind much of the labeling and ban legislation. Some participants expressed concern that cell-cultivated bans may restrict commerce or limit consumer choice, while others believed that the bans are “a huge distraction (P5)” and that consumers will ultimately determine product success. The state-by-state labeling approach is of concern as it “becomes really hard for new companies that are trying to get distribution, who are trying to meet this tangled web (P5),” explained one participant. They continued, “There’s a real need for streamlined labeling requirements that avoid confusion, avoid creating an unfair playing field for these new products, and just let them succeed or fail in the marketplace (P5).”

Stakeholder interviews were conducted shortly following the transition in federal government administrations in 2025. Stakeholders expressed uncertainty around how the U.S. policy landscape for AP products may shift with a new administration. They raised concerns around AP products becoming politically divided. Participants identified U.S. government tariffs on international trade, additional state or federal bans on cell-cultivated products, and an increased focus on UPFs as potential areas of uncertainty for APs amid a changing administration.

While stakeholders noted progress in AP policy and advocacy, many described the need for additional resources to support state and federal political engagement and education. Multiple stakeholders shared their belief that working with farmers and the incumbent industry will be critical for building political capital, and the sector could benefit from research on how to do so. Similarly, multiple participants expressed a need for additional research on the economic impact of the AP industry, including its impact on job growth, to help inform policymakers. Research on how to manage potential impacts on the incumbent industry is also needed, explained one stakeholder: “So alt proteins, I think, have really small impacts on livestock and crop producers today. Any group that has a vision for really substantial alt protein adoption, I think, really should meaningfully look at what would entail an effective transition? What are ways to engage with producers who might be affected negatively? How could policies or markets help minimize those negative impacts while still enabling the alt protein sector to grow (P20)?”

Many stakeholders described the need for continued government support to maintain U.S. AP leadership amid increasing investment from other countries. In particular, stakeholders saw China as a competitor in the AP space. For example, “How do we get the government to see this as a big opportunity to be a leader in the world? The U.S. has been the leader in this category—cultivated in particular—for the last 5 to 10 years. They’re about to give it up to China (P8).” Participants called for more research on the long-term health implications of AP consumption and the nutritional content of AP products. They believed that this research could inform future policy-relevant actions, such as incorporating APs into the U.S. Dietary Guidelines for Americans, which provide dietary recommendations for Americans and inform U.S. government policies and programs^27^. Federal and state procurement for schools and universities, military personnel, or government institutions was also identified as an opportunity space for the AP industry.

### Narratives

Consumer narratives were discussed by several stakeholders. A cross-cutting stakeholder sentiment was that consumers are not getting the right messages about APs, and the sector needs to improve how it communicates with consumers. As one described, “The best definition of ‘brand’ is ‘the promises you keep,’ and the alternative protein sector as a whole hasn’t quite figured out what that promise is, especially for those who don’t have a problem with eating meat (P6).” Early AP sector marketing narratives focused on APs as a replacement for conventional agriculture, encouraging a shift away from eating conventional meat and seafood products. Multiple stakeholders believed these narratives hurt the AP sector. One explained: “I think that approaching it as vegan or bust, everybody on the planet needs to go vegan, is not an approachable way to meet consumers where they are or to talk to consumers (P3).”

Others mentioned that, alongside achieving taste and price parity, APs need a value proposition, such as a nutrition or health benefit over conventional agriculture, as sustainability and climate benefits are not driving consumer purchase behaviors. According to participants, consumers also need more information on where to find AP products in stores, how to cook these products, and how to incorporate them into meals.

Looking toward the future, some stakeholders felt that the sector could benefit from a cultural marketing campaign to increase AP consumption. One likened it to the need for a campaign as effective as “Beef. It’s What’s for Dinner” or “The Incredible, Edible Egg” was for conventional agriculture. Some called for more research on how to market AP products to consumers. “I think the consumer needs to be at the front in many respects. What are they looking for? What are they interested in? What would entice them to try it? What would entice them not to try it? (P4),” one participant shared. Some AP sector stakeholders are working to shift the terminology from “alternative” to “complementary” proteins. A few participants shared that “alternative” sounds like “lesser than (P6)” or second-tier proteins, and it gives the impression these products are in competition with conventional proteins rather than offering another protein option to meet growing demand.

Policy narratives surrounding APs as UPFs were another particular concern. Multiple stakeholders shared their belief that the incumbent meat and seafood industries were behind narratives portraying APs as unhealthy. Some believed that the connection of APs to UPFs has already contributed to industry decline. Others expressed concern around these narratives’ potential future market impact, particularly amid bipartisan Congressional support for greater government action to address UPFs in the food supply. One shared, “We have an opportunity. We can either be in the non-processed, lightly processed area, or we can get thrown in with potato chips. And I think if we don’t advocate for ourselves right now, we’re going to be thrown in with potato chips, and I think that would be like the nail in the coffin (P2).” Participants described a need for more research on consumer perceptions of UPFs and on what messages may help consumers differentiate APs from other foods deemed ultra-processed.

Multiple stakeholders were concerned that APs may face similar narrative challenges as genetically modified organisms (GMOs), where concerns raised by some stakeholders led to widespread anti-GMO sentiments that were difficult to counter. For instance, “…There has been this sort of grassroots, anti-GMO blanket negativity in the U.S. for quite some time that holds a glorified view of food production where cows and lambs are frolicking in fields with buttercups—try doing that when you’ve got 350 million people to feed. I think the momentum around GMO is going to carry over to cultivated, and some sectors of the meat industry and segments of public opinion will try to vilify cultivated meat (P8).”

Many stakeholders expressed a need to tailor messages about the benefits of APs to Congressional priorities. Participants believed that messages focused on domestic food production, American job growth, economic development, national security, and U.S. competitiveness and leadership in the AP space would resonate with some legislators. As one participant shared, “We see incredible promise around really leaning in on [the] plant-based foods industry as a U.S. growth industry and the fact that if we don’t focus on this as a country, we’re going to get left behind by other countries, including China that are making big investments in plant-based (P11).” On seafood, one explained, “We import about 80%…If you eat salmon, it’s Atlantic salmon, it was grown on a farm, but a lot of fish just can’t be grown on farm. So, we’re actually not competing against the aquaculture industry. And if you say we can’t catch these fish in our domestic waters, okay we have to import it. So, you kind of get some conclusions here like, ‘Oh, it makes sense to make our own seafood’ (P10).” For other policymakers, the benefits of AP products on climate change and the environment, food security, and health and nutrition are relevant. A few participants believed that pro-AP arguments related to environmental sustainability or the climate are unlikely to resonate with the current administration. One expressed concern that branding AP products as environmentally sustainable alternatives could further politicize the sector.

Finally, several stakeholders discussed industry-facing narratives. When engaging with industry actors, particularly the conventional agriculture industry, multiple participants described adopting a more inclusive approach to talking about the role of APs in the food system. They described this as a “both/and” solution, in which APs complement conventional agriculture to meet the growing protein demand. One interviewee shared how they used this narrative to explain to conventional agriculture partners why their animal protein company was investing in APs. “We’re exploring this as an ‘and’ not an ‘or’ because the pie is growing,” they described. “The underpinning driver of this is the population is growing, and we have to feed more people with fewer resources (P12).” Another shared, “Stop your silly conversations about the competition with the conventional industry. We [have] got to feed the future generations (P1).”

### Stakeholder engagement dynamics

Focusing first on actors identified in our interviews as AP advocates, stakeholders shared that AP trade associations, such as the Association for Meat, Poultry and Seafood Innovation (AMPS) and the Plant-Based Food Association, were formed in recent years amid increased legislative and regulatory actions relevant to AP products. Some felt that trade associations are useful because they can “bring resources to everybody so that instead of us all separately trying to do the same thing, they’re able to galvanize everybody together in a way that I think is difficult otherwise (P12).” Yet multiple stakeholders shared their perspectives that AP trade associations have had little effect to date in advocating for or influencing government policy because, still in their infancy, these groups lack sufficient resources and bandwidth for widespread engagement.

Similarly, multiple stakeholders discussed NGOs’ work to educate government officials and encourage greater investment in AP R&D. For instance, one stakeholder shared how the Good Food Institute (GFI)—described by many as a leader in the AP space—was integral in securing a $5 million investment from California for AP R&D through the University of California, Davis, while Food Solutions Action helped secure R&D investment from the state of Massachusetts. GFI has also pushed back against state-level labeling and ban legislation. Yet others noted that GFI’s non-profit status limits its capacity to engage in policy and lobbying. A few believed that global health and sustainability/environmental organizations could be doing more to advocate for AP products. While public universities cannot engage in lobbying, stakeholders described their efforts to educate state and federal government officials on AP products as important for the sector.

According to participants, the media could have either a positive or negative impact on an AP product’s success. Social media influencers, in particular, were seen as potentially impactful drivers of consumer AP adoption, although participants did not name specific influencers. Registered dietitians, nutritionists, and the medical community were similarly identified as relevant AP influencers, particularly for cell-cultivated seafood, given its health benefits over traditional seafood (e.g., lower mercury levels). As one cell-cultivated seafood startup explained, “Who is your influencer? It’s not some celebrity…It’s actually a chef or a nutritionist or doctor (P1).”

Chefs and the culinary community could also support AP adoption by showing people how to prepare APs in a tasty way and as part of their regular diet. Participants believed that retailers and others in the food service space are similarly influential. “I think there’s a lot of opportunity for this sector to engage retailers and food service operators, buyers who hold a lot of power in food trends (P6),” one participant said. “And quite frankly, no one can eat these products if they’re not put on the shelf or on a menu, and they’re just people making those decisions (P6).”

On the other hand, stakeholders saw the meat and dairy industries, particularly industry trade associations, as primary AP challengers that “have the ear of quite a few members of Congress (P11).” For example, “I think that…stakeholder groups — especially agricultural organizations, trade associations, commodity groups — have long been able to drive up opposition and concern from policymakers about alternative proteins by claiming that these products will be ruinous for them (P20).” Similarly, some stakeholders felt the incumbent meat and seafood industry has influenced negative consumer perceptions by, for instance, “seeding doubt in consumers’ minds that these are not the healthy products that they thought they were (P18).” One participant mentioned research on narratives that shows the pharmaceutical industry has funded anti-AP messaging, likely because the industry benefits from high antibiotic use in conventionally farmed animals.

Yet some conventional industry actors have diversified their portfolios to include APs and may therefore be more accepting of these products. One stakeholder explained that some conventional agriculture companies had a “framing shift several years ago from saying, ‘We’re meat companies,’ to saying, ‘We’re protein companies’ (P17).” They went on to express, “I think to the extent that that’s genuine, they could be massive forces for good in accelerating this industry. To the extent that that’s not genuine and that they see this as a threat and they’re not interested in getting involved in alt proteins, they can be among the most challenging needle movers of the sector (P17).”

For the AP sector to succeed, multiple participants expressed that collaboration is crucial. They shared that start-ups must partner with the incumbent industry to see sizeable action in shifting the U.S. food system because “They have the scale, they have the capital, they have the expertise, they have the brand recognition and consumer trust. They have the distribution partnerships; they have the leverage (P17).” Legacy meat and seafood industry actors, on the other hand, can benefit by addressing issues in their current production processes and expanding their product portfolios. Greater collaboration and knowledge sharing across the AP sector was further identified as necessary to achieve taste and price parity and bring innovative products to market. Another expressed that cross-sector collaboration, supported by interdisciplinary AP research centers, can help move from a siloed R&D approach based on AP type to “start to integrate those technologies and really ask questions like, ‘what are the best starting materials and ingredients to make the best meat analog that meets price and taste goals?’ and not be constrained by just working with plant-based materials (P18).”

### Role of APs in the future of food

Food security, particularly the need to feed a growing global population, arose as a common theme in stakeholders’ visions for the future food system. Stakeholders commented that current meat and seafood supply chains cannot meet the growing global demand for protein products in the coming decades. They felt that APs can help meet this need. As one shared, “The question ultimately is, how do you feed a world of soon to be 10 billion people sustainably? And I think alternative proteins, alongside a whole set of other solutions, are part and parcel of that package (P5).” In the short term, participants expressed that AP products are likely to play a greater role in the food systems of higher-income countries compared to those of lower-income countries.

A few stakeholders noted that APs could be more resilient to supply chain shocks than conventional agriculture. For instance, they expressed that these products may offer consistency in the food supply chain by mitigating complex issues related to climate change, overfishing, zoonoses, geopolitics, and trade, among others, and may offer better health outcomes. A few saw APs as the future of meat and seafood, but most believed APs will be an additional offering alongside—rather than a replacement for—conventional agriculture products, harkening back to the “both/and” narrative.

However, one participant said that, for AP products to grow in market share, Congress and the food industry must prioritize addressing shortcomings in the current food system, rather than “put a Band-Aid on it for another 15 years (P2).” As they described, “If we do things right, APs have the potential to be 10% of the marketplace in 10 years. It has the possibility to be dead this year. It just depends on how you play your cards (P2).” Similarly, stakeholders shared the need for greater consumer acceptance to foster AP market growth.

Future AP innovations offer unique opportunities for new products. As one stakeholder said, “Over, say, the next couple of centuries, I would expect that the types of food that we eat will change dramatically because these technologies fundamentally afford us a lot more. We’re painting with a new palette of colors here…We can build products from the ground up to be what we want rather than mimicking what came before (P15).” However, this is not expected to occur quickly, but rather over decades, with the same participant describing it as “a very slow-moving revolution (P15).” As another shared, “I can definitely imagine a future state where technologies that were originally designed to produce meat and seafood analogs are then translated into other parts of the biomanufacturing industry just because of the innovation that’s happened (P18).”

## Discussion

The AP stakeholders interviewed for this study described the sector as rapidly evolving. Yet, while many leaps in innovation have occurred since the initial AP boom of 2009–2021, stakeholders noted ongoing challenges on the commercialization pathway. Below, we draw on existing scientific and practitioner-based innovation frameworks to reflect on the AP sector’s evolution. We also explore implications of the current market, consumer, and policy challenges on the sector’s future. Findings from this reflection may be useful for identifying research and innovation priorities that could impact the future of the AP sector.

The AP sector’s evolution, as described by interviewees, seems to follow a well-established pattern of innovation. One framework, the GHC^28^, was mentioned by one participant. It has been used by academics to describe the cellular agriculture sector^29^, and multiple media articles have used the GHC to describe the overall AP sector^30,31^. This innovation cycle was created by the research and advisory firm Gartner but has been used to describe other innovations within academic literature as well^32,33^. The GHC starts with an initial “innovation trigger” that sparks rapidly growing interest. For the AP sector, this was the “boom” in the popularity of products like Beyond Meat and Impossible Foods burgers, as well as the development of the first cell-cultivated burger, in the early 2010s (Fig. 1). Early successes built to a “peak of inflated expectations,” which corresponds to the 2021/2022 bubble, when U.S. AP sales reached an all-time high and began to decline^8^. When initial feats were not matched in successive performance, there was a decline into what Gartner calls the “trough of disillusionment,” corresponding to participant descriptions of constriction and consolidation in the sector. Following the GHC, innovations can eventually ascend a steadier “slope of enlightenment” towards the “plateau of productivity,” when they have matured enough to become mainstream. While some stakeholders believed that the AP sector was still in a challenging stage as of early 2025, indicating the sector had not left the “trough,” one stakeholder felt the sector was recovering, signaling potential progression towards the “slope of enlightenment.”

Our application of the GHC is relevant, as it is the prevailing innovation framework used among stakeholders and in the media to describe the AP sector. Yet, some researchers have raised concerns about the GHC’s applicability and inconsistencies^34^. Looking at more established academic theories of innovation, few have been applied to the AP sector. One notable example is Bulah et al. (2023), who applied the technological innovation systems framework in combination with institutional work theory to assess the rise of the U.S. plant-based AP sector^35^. The authors assessed the sector up to 2019, and the framework aims to explain the trajectories of various innovations rather than providing a template for the trajectory of innovation, limiting comparison with our analysis. However, the authors concluded that by 2019, plant-based APs had successfully diffused the dominant meat regime in the U.S. An extension of this research is needed to determine whether these theories could explain the decline in the AP sector that followed 2019. Overall, we find that the U.S. AP sector provides a case rich in data that could advance or test established theories of innovation, and further research is needed. Regardless, the AP stakeholders we interviewed believed that more work is needed to reach mainstream diffusion. As such, we provide a deeper analysis of major AP sector challenges—based on stakeholder interviews and highlighted in recent developments—and identify further research that could move the sector forward.

From a consumer perspective, achieving parity in price and taste remains a key ongoing challenge. A 2025 study by GFI found that only 15% of U.S. consumers associated plant-based meat with being “affordable” and only 21% found it “tasty.”^36^ Yet some participants noted that to achieve consumer acceptance, AP products should surpass parity and be less expensive than conventional animal-based counterparts. Several recent studies support participants’ suggestions that blended/balanced proteins can achieve taste parity^37^. A sensory panel of over 1000 U.S. omnivores and flexitarians reported that balanced burgers and nuggets (i.e., “products that blend conventional animal protein with a significant proportion of plant-based ingredients”) performed better than their animal-only counterparts in blind taste tests^38^. Similarly, an AP-enhanced chicken mince outperformed conventional chicken mince in a blind consumer panel in Singapore^39^, findings which could be useful for identifying opportunities to test these products with U.S. consumers. Future research could explore consumer acceptance of novel AP products identified by stakeholders, such as balanced/blended protein products or APs with international flavors. Additionally, new ingredient development could enhance the flavor profile and/or reduce AP product costs^40^. Research and innovation are also needed to create new high-throughput production methods—and advance existing ones, including fiber spinning, 3D printing, extrusion, and forming^41–44^—to enhance sensory attributes and/or reduce costs, as mentioned by participants.

From a market perspective, stakeholders consistently raised the challenge of achieving scale. Key hurdles identified both by stakeholders and in the literature include high capital and operational costs^45^, lack of shared test facilities^46^, challenges of moving from lab to large-scale operations^47^, price parity with conventional meat products, and lack of regulatory guidance on processing requirements. Large investments^48^ and interdisciplinary collaboration^49^ will be necessary to overcome these challenges. Cell-cultivated meat, as the most recent addition to the AP landscape, faces the greatest challenges to scale-up. Innovations are actively being developed, however, to achieve cost-effective scaling of cell-cultivated APs. For instance, advancements in cell lines, media, scaffolds and bioreactors show promise in reducing production costs^49^. Despite these advances, more research and innovation are needed^49–51^ including development of low-cost, scalable, food-grade culture systems; optimized bioreactor design; advanced integration of supply-chains and industrial infrastructure; and effective methods to increase consumer acceptance.

From a policy perspective, the UPF conversation has gained momentum. Participants were aware of the potential pushback against UPFs, citing discussions like the 2024 congressional hearing titled “What is the FDA Doing to Reduce the Diabetes and Obesity Epidemics in America and Take on the Greed of the Food and Beverage Industry”^52^. At the center of this debate was the role of “addictive” UPFs in promoting obesity and diabetes^53^. Since our interviews in July 2025, the U.S. government released a public request for information to inform the development of a definition for UPFs in the U.S. food supply to guide research and policy actions^54^. In October 2025, California enacted the first state-level law banning UPFs in school lunches^55^. Shortly after, in December 2025, the city of San Francisco filed a complaint against 10 food companies accused of developing, aggressively marketing, and selling harmful and addictive UPFs, particularly to children^56^. The definition adopted by policymakers may impact future AP regulation and lawsuits, availability in the U.S. market, and consumer perception of these products. While there is currently no widely agreed-upon definition for UPFs, many prominent researchers use the NOVA classification to define any food made with industrial processes or additives as an UPF^57^. Under this definition, some plant-based APs, including plant-based meat alternatives, are classified as UPFs^58–60^. It is unclear how the NOVA classification addresses novel technologies like cell-cultivation. Some policymakers, however, have adopted a more nutritive-focused definition, presenting UPFs as junk foods high in sugar, salt, and/or unhealthy fats^52^. Certain plant-based meats can be high in sodium^61^, thus qualifying them as UPFs using “nutritive” definitions. Future research could clarify the relationship between UPFs and APs. For example, despite being labeled as UPFs, research has shown that plant-based APs generally have a better nutrient profile than the foods they are meant to replace and are not associated with certain health risks associated with animal-based UPF foods^10,62^. Additional research could explore the long-term health implications of APs to determine whether APs warrant an exception to the UPF classification. Future research could also explore consumer perceptions of APs as UPFs, identifying nuanced narratives that help consumers differentiate between nutritionally-poor UPFs (e.g., candy and chips) and health-promoting AP products that may offer sustainability benefits. Additionally, stakeholders stressed the need for additional research into state-level economic analyzes of the AP sector and the implications of AP sector growth on legacy agricultural producers advance policy discussions.

The findings of this paper should be interpreted with the following limitations and considerations in mind. First, as previously mentioned, although we reached out to individuals and entities publicly expressing less favorable views of AP products, none agreed to participate. Inclusion of more diverse stakeholders could have identified additional opportunities and challenges for the U.S. AP sector. Similarly, despite reaching out to several stakeholders focusing on fermentation-based AP products, relatively few agreed to participate. Inclusion of such actors could have provided additional insights for the fermentation-oriented AP sector. That said, several stakeholders focused on all AP types in their work, and the relatively small discussion of fermentation-based products could accurately reflect the trends in the U.S. AP sector. While we included measures to prevent selection biases (see “Methods”), some stakeholders might have been more or less willing to participate knowing that the research team was conducting this analysis on behalf of a center for sustainable proteins. Finally, some invited participants might have declined to participate due to the timing of the interviews — during the early part of 2025 in the U.S. — due to perceived sensitivities. Nevertheless, the stakeholders interviewed are considered key players in the U.S. AP sector, and their insights are well-informed and helpful when considering the state of the sector. Similar research could explore whether the AP sectors of other countries and regions show similar trends, opportunities, and challenges, given that this research cannot be generalized beyond the U.S.

Despite the challenges facing the sector, the stakeholders we interviewed were generally optimistic about AP products’ future role as a complement to conventional meat and seafood products. As noted, none of the participants who agreed to take part in this study publicly opposed APs. Different visions for the AP sector’s future may have emerged had entities with less supportive views of AP products agreed to participate. Additionally, the U.S. AP sector is not developing in a vacuum, and its future trajectory will be influenced by actions and innovations elsewhere in the world. While it was beyond the scope of this paper, other countries have made significant investments in APs, enacted supportive policies, and fast-tracked approval processes for these products^63^. It will be important for U.S. researchers and AP stakeholders alike to keep abreast of international developments. Additionally, stakeholders stressed the need for additional research into state-level economic analyses of the AP sector as an important tool in policy discussions. Similarly, research on the implications of AP sector growth on legacy agricultural producers, as well as collaboration with legacy agricultural producers, could advance U.S. policy discussions. At present, the U.S. AP sector is a rapidly evolving field at a critical juncture in its development, and one facing both challenges and opportunities as it works to scale up production to meet growing protein demands. By identifying and describing expert stakeholder views on the AP sector’s present landscapes and future needs, this study offers a strong foundation for informing the sector’s strategic path forward.

## Methods

This qualitative study consisted of a thematic analysis of stakeholder interviews. Researchers sought ethical approval through the Duke University Campus Institutional Review Board to conduct stakeholder interviews. Ethics approval was obtained in December 2024 (Protocol ID #2025-0222), and all methods were performed in accordance with this protocol, the Declaration of Helsinki, and other relevant ethical guidelines and regulations. According to the ethical protocol, only researchers at Duke University (AW; KCS; JD; NLWW) were authorized to engage with participants and analyze data; that is, participant identification and information, interview recordings, and transcript access were limited to the Duke University research team. AW also obtained ethics approval from the national ethics authority in Sweden (Etikprövningsmyndigheten – ID# 2025-03681-01-779985) to process and analyze deidentified data while working in Sweden, and all work was carried out in accordance with the relevant national regulations and the Declaration of Helsinki. Collaborating authors from North Carolina State University contributed to the study, as documented in the CRediT statement, but we note that they did not have access to participant identities or data collected from participants to adhere with the IRB protocol.

### Stakeholder identification

Potential interviewees were identified through a stakeholder analysis of multiple sources. First, Google was used to search for potential interviewees in the U.S. using terms relevant to APs (e.g., combinations of AP terms such as “alternative meat”, “cell-based meat”, “plant-based seafood”, etc. and “United States”). Ninety-four grey literature documents from U.S. stakeholders were collected, including reports, press releases, fact sheets, articles on stakeholder websites (excluding media articles), regulatory frameworks, and roundtable reports, from which we extracted relevant stakeholders.

Second, we searched industry newsletters, given the rapidly evolving nature of the sector. Food Navigator (U.S.) articles from Jan 1, 2024 to Dec 31, 2024 were searched using the phrase ‘alternative protein.’ In total, 153 articles were relevant to alternative meat and seafood, and AP stakeholders were identified from these articles.

Third, we hand-searched the GFI website (U.S.), collecting 42 documents, including reports, fact sheets, white papers, partner resources, and technical summaries. Additionally, GFI provides an open-access database of AP companies^64^. We downloaded the database on October 30, 2024 and narrowed the businesses to those headquartered in the U.S. with a “company focus” that included meat and/or seafood. Within these parameters, 210 companies were included. Finally, from GFI’s AP researcher directory^65^, we included laboratories, research groups, and researchers from North America who focused on AP meat or seafood products.

Fourth, to ensure we were not missing other key actors, we manually searched for U.S. AP federal regulators. Fifth and finally, we used a list of key stakeholders that had been pooled together by North Carolina State University researchers at the Bezos Center for Sustainable Protein to contribute to our list of potential participants.

### Stakeholder selection

The stakeholder analysis detailed above identified hundreds of potential stakeholder participants. To narrow the list of potential interviewees, we developed a stakeholder matrix to ensure representation across sectors (Table 2). Potential stakeholders were limited to individuals who worked with APs in a professional capacity.

**Table 2:** AP stakeholder matrix used to categorize potential stakeholder interviewees in the study

	Policy & government	Companies	Industry association	Investors	Non-governmental organizations	Research
**Support**						
Supporter						
Detractor						
**AP products**						
Meat						
Seafood						
General/ both						
**AP type**						
Plant-based						
Fermented (all types)						
Cell- cultivated						
Mix/all						
**Interest**						
Consumer protection						
Policy and Regulation						
Market (profit)						

Using this matrix, we developed a shortlist of potential participants, which was discussed and refined. While it was not possible to interview a participant representing each matrix cell, we ensured that at least one potential participant was identified in every row of the matrix.

Researchers at Duke University invited 38 stakeholders to participate in the research study via email in December 2024, following IRB approval. Follow-up emails were sent in early January. Interviews began in January 2025. At the end of the interviews, participants were asked to forward the research team’s contact information to other potential participants, using a snowball approach to identify other potential study participants (see Supplementary File 1). These individuals were instructed to contact the Duke University research team if they wished to participate in an interview. Four additional stakeholders were emailed in April 2025 to request participation in the study.

### Interviews

Semi-structured interviews were conducted via Zoom and lasted an average of 48 min and 1 s. The interview guide is presented in Supplementary File 1. Written informed consent was obtained from all participants prior to the interviews. Interviews were audio recorded and transcribed. Transcriptions were then deidentified for analysis. In the consent form, participants could choose whether direct quotes from the interview could be used, and four declined to be quoted directly (P7, P9, P14, P21).

### Interview analysis

Deidentified transcripts were analyzed by two coders using NVivo (release 14.24.3) following the six-step framework for thematic analysis by Braun and Clarke^66^. Phase one requires coders to familiarize themselves with the data. Both coders were deeply familiar with the transcripts, since the transcripts previously informed the writing of an internal report. To generate initial codes (Phase 2), a two-level codebook was developed, agreed upon, and used to code all transcripts. To ensure coding consistency, coders co-coded 20% of the transcripts (*n* = 4) and conducted an inter-rater reliability test producing a Kappa of 0.85, signaling excellent agreement. Both coders met remotely to review the coded extracts, search for potential themes (Phase 3), check if all coded extracts were accounted for in a theme (Phase 4), and define and refine the themes (Phase 5). After finalizing the themes, Phase 6 consisted of writing up the themes, which are presented in the results.

## Supplementary information

Supplementary File 1

## Data Availability

The data generated during and/or analyzed during the current study are not publicly available to protect the anonymity of participants. De-identified, aggregated data may be available from the corresponding authors on reasonable request, if the request adheres to the ethical requirements set out by the Duke University Campus Institutional Review Board and the terms of this study’s protocol (Protocol ID #2025-0222).
