# Supplementary material for: Trends, challenges, and opportunities for the United States alternative meat and seafood sector: stakeholder-informed perspectives
Source: NPJ Sci Food. 2026 Apr 17;10:133. doi: 10.1038/s41538-026-00841-4 (PMC13090389; doi:10.1038/s41538-026-00841-4)
Supplement: Supplementary file 1 [file 41538_2026_841_MOESM1_ESM.docx]

**Supplementary File 1 – Stakeholder interview guide**

1. Please briefly summarize your connection to the alternative protein space and experience in the sector.

1. Looking back over the past few years, what key trends have you been observing relative to the alternative protein sector? *In particular, market, consumer, or policy trends.*
2. Looking ahead over the next 5-7 years, what opportunities do you see within the consumer, market or policy landscapes for the development of the alternative protein sector?
3. Again, looking over the next 5-7 years, what threats or barriers do you see emerging in the consumer, market or policy landscapes to the successful development of the alternative protein sector?

1. In what ways do you think the alternative protein sector is excelling and in what ways do you think it needs improvement?

1. Are there specific areas, challenges, or issues of alternative proteins that you feel are being overlooked or undervalued?

Now we are going to focus on key stakeholders or players in the alternative protein sector.

1. Who would you say are the most influential players that will impact consumer adoption and market growth either positively or negatively for alternative proteins and why?

*(Probe: That could be across industry, regulators, celebrity chefs, advocacy or anti-advocacy groups etc.)*

1. What type and level of influence do you think [NAME OF THEIR ORGANIZATION OR COMPANY] has in the alternative protein space?

1. What advocacy efforts exist in this space for or against alternative proteins?

*(Probe: Are these efforts effective? Who are the key players driving them?)*

Now we will shift to various aspects of research.

1. What areas of research do you think are most needed over the next 5-7 years to advance the alternative protein sector?

(*Probe: For example, research to scale-up production of alternative proteins)*

1. What kinds of activities, initiatives, and/or products would be most beneficial to the alternative protein space coming out of an interdisciplinary research center on alternative proteins, such as the new Bezos Center for Sustainable Proteins?

(*Probe: For example, research briefs, published papers, workshops, collaborative research projects with industry*)

1. What types of engagement would be most effective for the Bezos Center to create a two-way flow of information between academia and relevant practitioners?

*(Probe: For example, workshops, focus groups, conferences, advisory panels, collaborative research projects, etc.)*

1. Looking big picture - what do you see as the role of alternative proteins in the future of food?

We have two final questions before we wrap up.

1. Are you open to a follow up interview in the future? (state potential purposes: e.g., it might be helpful to ask for additional information that may have been missed in this interview)

1. Based on the questions I asked you today, do you think there are other key stakeholders we should speak with to gain insights into the alternative protein space? *Before you answer, if you have suggestions, we cannot collect personal information such as names directly from you, but you can either give us the name of their organization and department, or you can forward the contact information of the research team to the individual and ask them to get in touch with us.*

THANK YOU AND WRAP UP
